# Supplementary material for: Tetrathiomolybdate induces dimerization of the metal-binding domain of ATPase and inhibits platination of the protein
Source: Nat Commun. 2019 Jan 14;10:186. doi: 10.1038/s41467-018-08102-z (PMC6331642; doi:10.1038/s41467-018-08102-z)
Supplement: Supplementary file 1 — Supplementary Information [file 41467_2018_8102_MOESM1_ESM.pdf]

Supplementary information

Tetrathiomolybdate Induces Dimerization of the Metal-binding Domain of ATPase and Inhibits Platination of the Protein

Fang et al.

Supplementary Figures

ATP7B MBD1-6 sequences :

```
 56  60         70        80        90        100       110       120
  QVATSTVRILGMTCQSCVKSIEDRISNLKGIISMKVSLEQGSATVKYVPSVVCLQQVCHQIGDMGFEASIA

   130       140       150       160       170       180       190
  EGKAASWPSRSLPAQEAVVKLRVEGMTCQSCVSSIEGKVRKLQGVVRVKVSLSNQEAVITYQPYLIQPEDL

  200       210       220       230       240       250       260
  RDHVNDMGFEAAIKSKVAPLSLGPIDIERLQSTNPKRPLSSANQNFNNSETLGHQGSHVVTLQLRIDGMHC

 270       280       290       300       310       320       330
  KSCVLNIEENIGQLLGVQSIQVSLENKTAQVKYDPSCTSPVALQRAIEALPPGNFKVSLPDGAEGSGTDHR

340       350       360       370       380       390       400       410
  SSSSHSPGSPPRNQVQGTCSTTLIAIAGMTCASCVHSIEGMISQLEGVQQISVSLAEGTATVLYNPSVISP

         420       430       440       450       460       470       480
  EELRAAIEDMGFEASVVSESCSTNPLGNHSAGNSMVQTTDGTPTSVQEVAPHTGRLPANHAPDILAKSPQS

        490       500       510       520       530       540       550
  TRAVAPQKCFLQIKGMTCASCVSNIERNLQKEAGVLSVLVALMAGKAEIKYDPEVIQPLEIAQFIQDLGFE

       560       570       580       590       600       610       620
  AAVMEDYAGSDGNIELTITGMTCASCVHNIESKLTRTNGITYASVALATSKALVKFDPEIIGPRDIIKIIE

      630
  EIGFHASLAQ
```

MNK3 sequences :

```
275                                                                    346
  NDSTATFIIDGMHCKSCVSNIESTLSALQYVSSIVVSLENRSAIVKYNASSVTPESLRKAIEAVSPGLYRVS
```

Supplementary Figure 1 The protein sequences used in this work. The following domains were constructed for assays: WLN4 (aa 357-428), WLN5 (aa 486-557), WLN6 (aa 562-633), WLN1234 (aa 56-432), WLN456 (aa 357-633), and MNK3. The Cu-binding motifs CXXC were highlight in red. The metal binding domains (MBDs) were underlined.

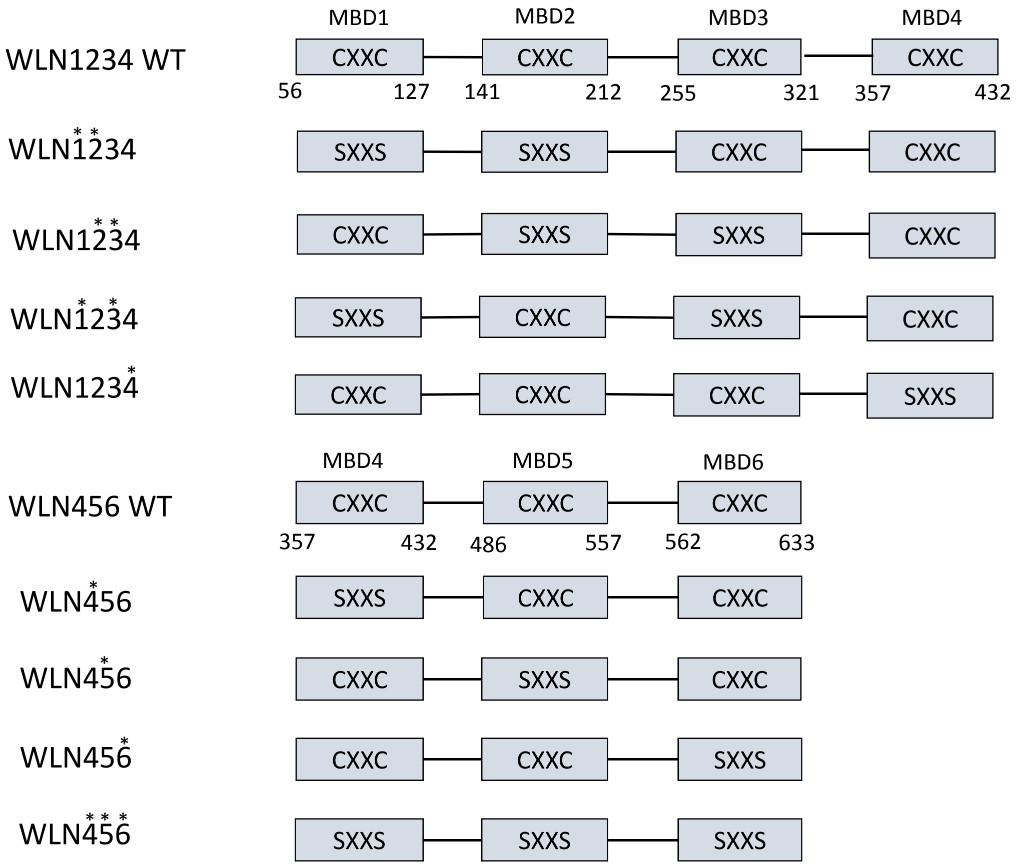

Supplementary Figure 2 Schematic illustration of multi-domain proteins of ATP7B and mutants used in this work. The cysteine residues in the metal binding motif (CXXC) were replaced by serine in different mutants (SXXS). WLN1234 represents the domain 1-4 of WLN protein, and WLN456 represents the domain 4-6 of WLN protein. The asterisks denote the mutated domains.

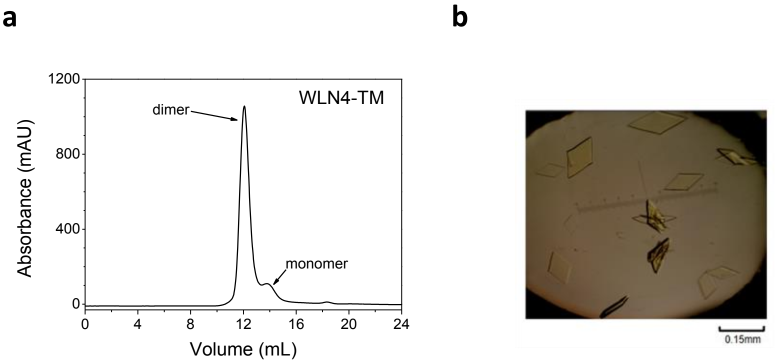

c

Cu	Mo
0.000 μM	0.039 μM

Supplementary Figure 3 Sample preparation. **a** Gel filtration chromatogram for purification of Cu/WLN4-TM. The sample was prepared by incubation of 0.4 mM Cu-WLN4 with 1.2 mM TM. **b** Crystals of TM-WLN4 obtained by incubation of TM with Cu-WLN4. The scale bar (0.15 mm) is given blow the figure. **c** The amount of Cu and Mo in the crystal of TM-WLN4 obtained from the reaction of Cu-WLN4. The crystals were dissolved and the contents of Cu and Mo in the solution were analyzed using ICP-MS.

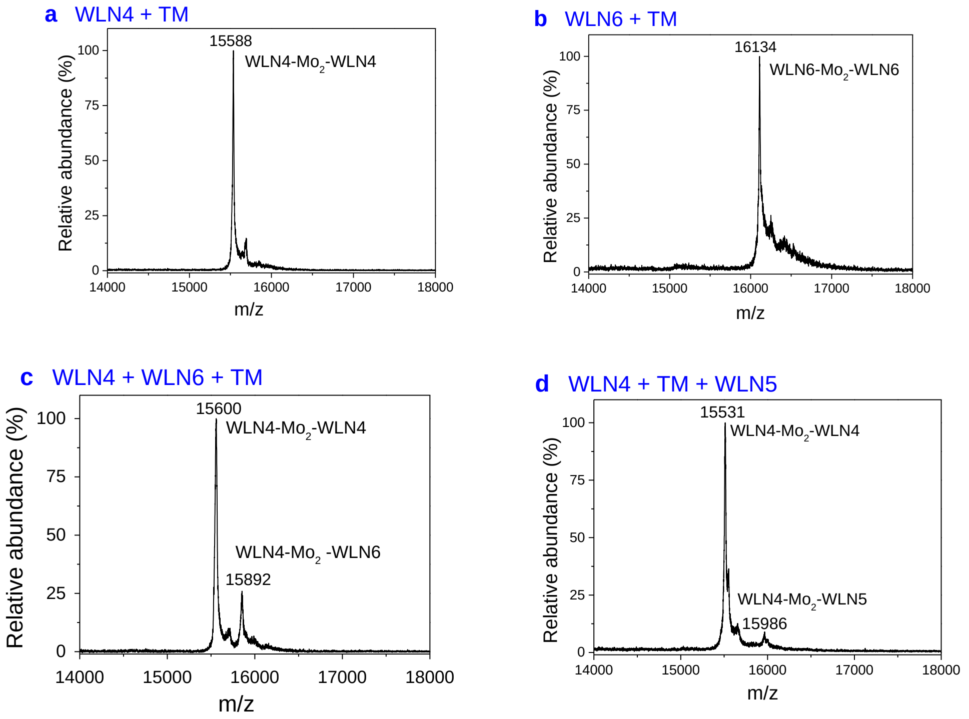

Supplementary Figure 4 Mass spectrometric analysis of the dimerization induced by TM. **a** WLN4; **b** WLN6; **c** the mixture of WLN4 and WLN6 (1:10 molar ratio). Peaks were assigned to WLN4-TM-WLN4 (m/z 15600); WLN4-TM-WLN6 (m/z 15892). **d** the mixture of WLN4 and WLN5 (equimolar). Peaks were assigned to WLN4-TM-WLN4 (m/z 15531); WLN4-TM-WLN5 (m/z 15986). All reactions were performed at 37 °C for 3 h. The peak assignments were listed in Supplementary Table 2.

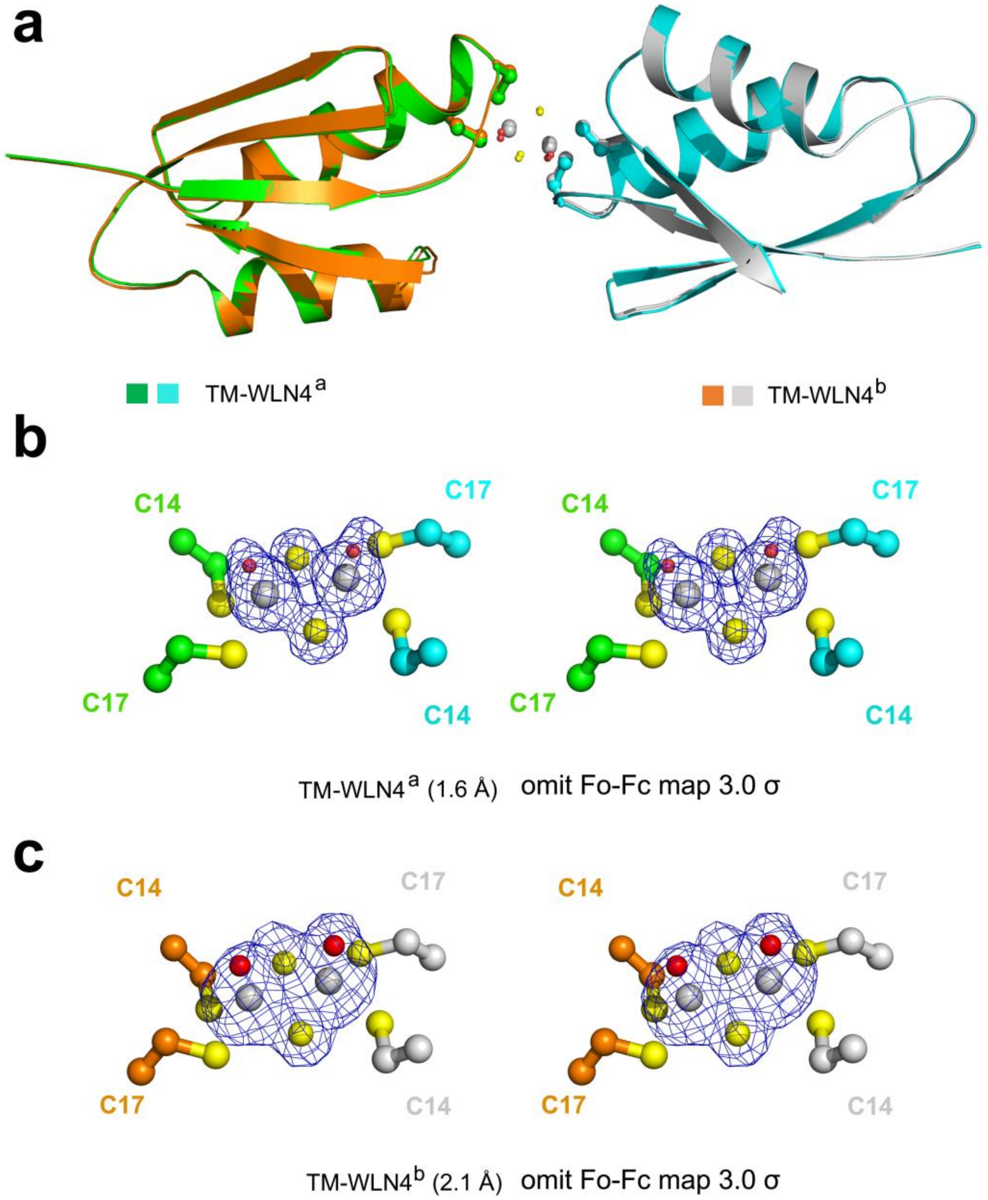

Supplementary Figure 5 Comparison of the crystal structures of TM-WLN4 dimers prepared by Cu-WLN4 (TM-WLN4[a]) or apo-WLN4 (TM-WLN4[b]). **a** Superposition of the overall dimeric structure of TM-WLN4[a] and TM-WLN4[b]. The two WLN4 subunits are colored green and cyan for TM-WLN4[a], orange and gray for TM-WLN4[b], respectively. The Mo, S and O atoms are shown as sphere and colored gray, yellow and red, respectively. **b, c** Stereoview of the omit *Fo-Fc* density map of $Mo_2S_2O_2$ group in TM-WLN4[a] (**b**) and TM-WLN4[b] (**c**) structures are contoured at 3 σ.

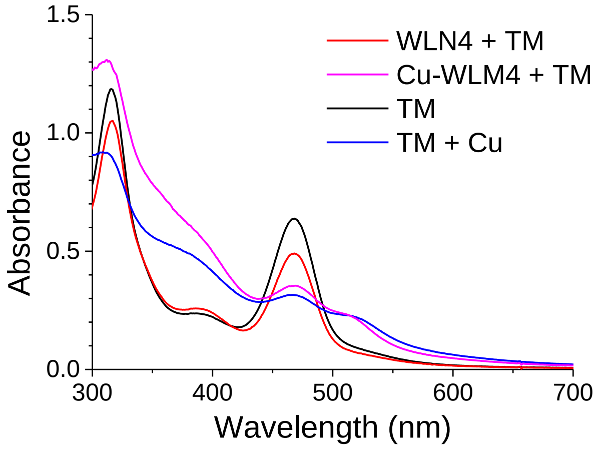

Supplementary Figure 6 UV-vis spectra of TM in the reaction with copper ions or WLN4. The spectra recorded on reaction of TM with apo-WLN4 (red) or Cu-WLN4 (magenta). The reactions were performed on 60 µM TM with 30 µM protein at 37°C for 2 h. The spectra of 60 µM TM in the absence (black) or in the presence of Cu(I) ions (blue). Upon Cu(I) reaction, the characteristic peaks of TM at 317 nm and 468 nm decreased, while two shoulder peaks appeared at ~400 nm and 520 nm from the Cu-TM complex.

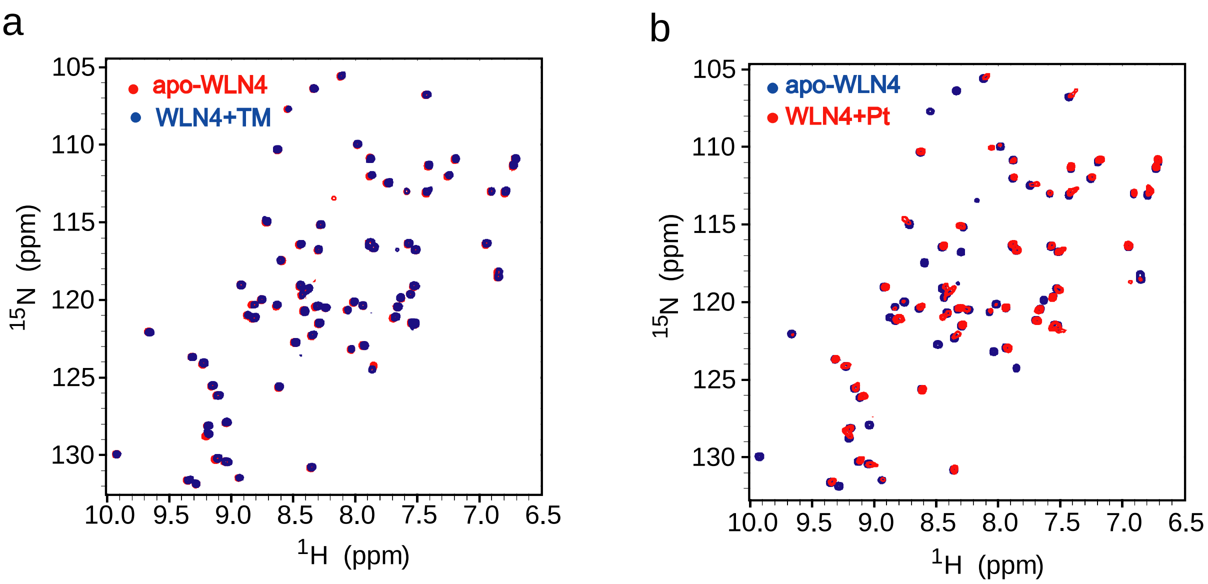

Supplementary Figure 7 ^{1}H-^{15}N HSQC NMR spectra of WLN4. **a** Spectra overlay of apo-WLN4 in the absence (red) and presence (blue) of TM. 3 molar eq. of TM was added to apo-WLN4. **b** Overlay of apo (blue) and cisplatin-bound (red) wild-type WLN4 spectra. 0.5 mM WLN4 was treated with 1.5 mM cisplatin at 37 °C for 10 h.

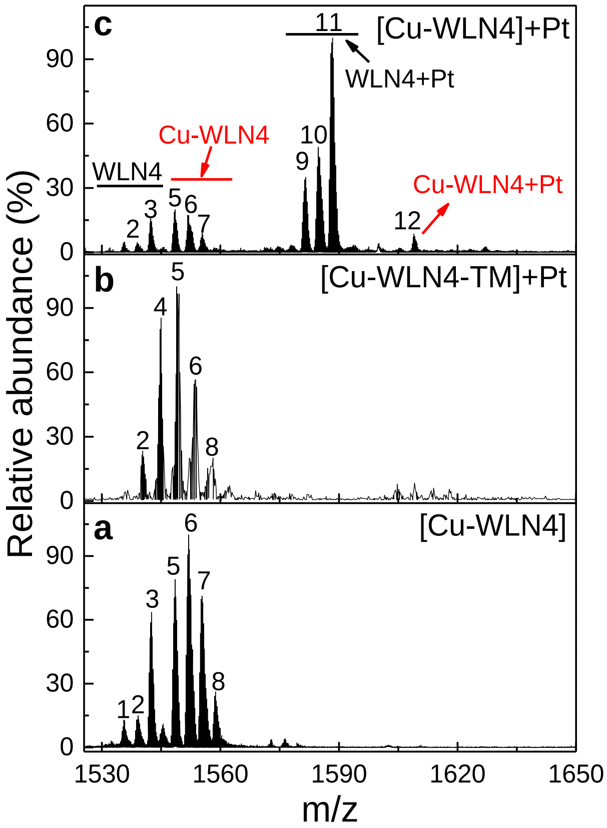

Supplementary Figure 8 ESI-MS analysis of the effect of TM on the platination of WLN4. **a** Cu-WLN4; **b** the reaction of cisplatin with TM/Cu-WLN4 adducts; **c** the reaction of cisplatin with Cu-WLN4. The platination reaction was conducted on 50 μM protein with 150 μM cisplatin at 25 °C for 8 hours. The selected m/z region shows +5 charged peaks. NH_4OAc was added to the samples before detection. The peaks are shown in a cluster due to the binding of different number of NH_4^+ ions. The +5 charged peaks are labelled in spectra and the species were listed in Supplementary Table 3.

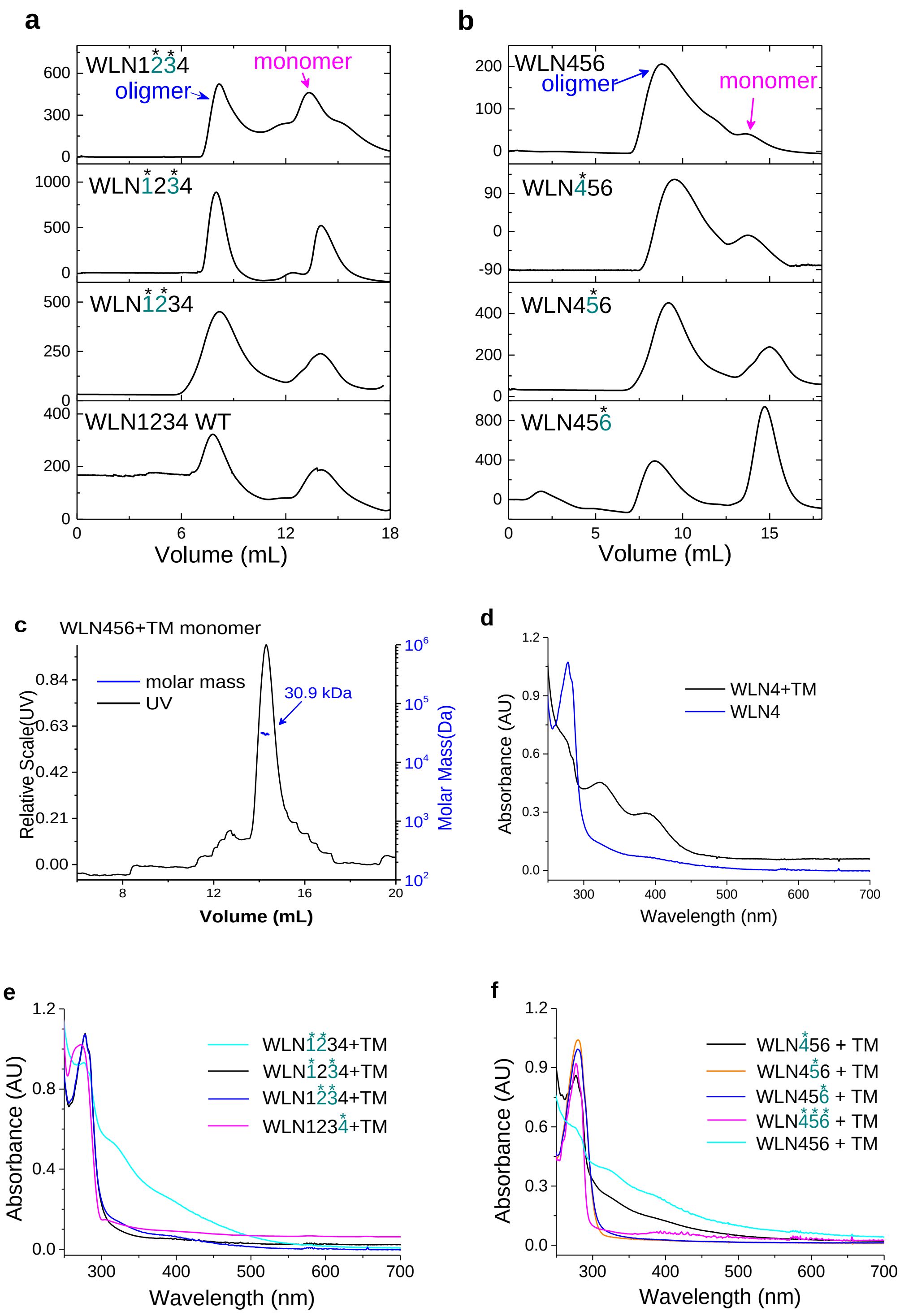

Supplementary Figure 9 Reaction of TM with multi-domain proteins. **a** SEC results of WLN1234 and its variants; **b** SEC results of WLN456 and its variants; **c** SEC-MALS analysis of the monomeric species obtained from the reaction of WLN456 with TM; **d** UV-vis spectra of WLN4 and the WLN4-TM adduct; **e, f** UV-vis spectra of the TM adducts of multi-domain proteins (**e**: WLN1234, **f**: WLN456). All adducts from multi-domain proteins were purified by SEC, and the fractions of monomeric species were collected for UV-vis analysis. Asterisks denote the mutated domains in which the metal binding site CXXC was replaced by SXXS.

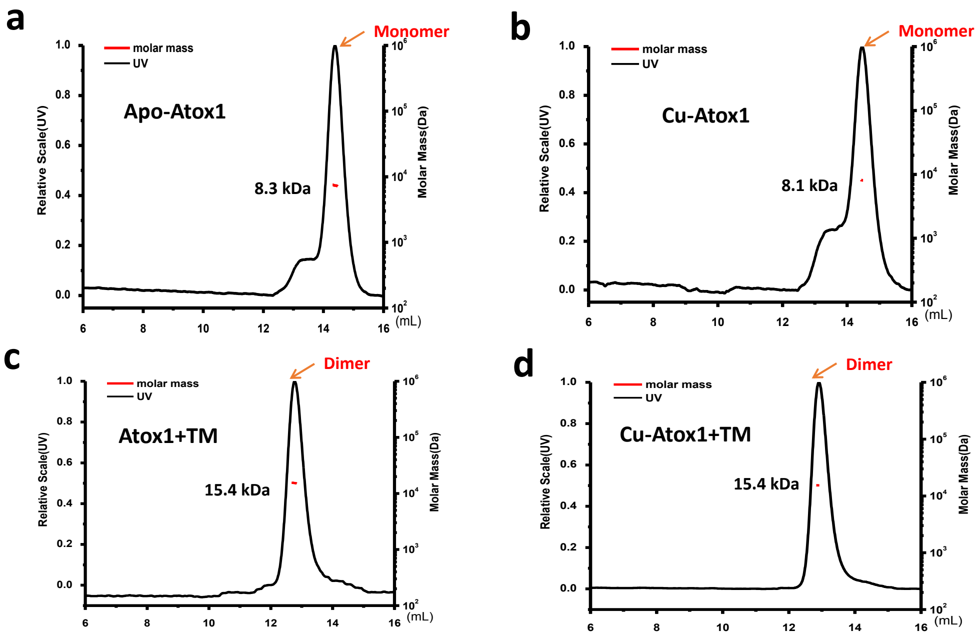

Supplementary Figure 10 SEC-MALS measurements of Atox1 and its TM adduct. **a** Apo-Atox1, **b** Cu-Atox1, **c** apo-Atox1 incubated with TM, **d** Cu-Atox1 reacted with TM. The calculated molecular weight and oligomeric state of each sample is labeled black and red, respectively.

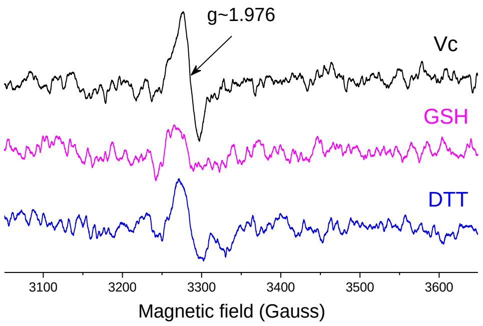

Supplementary Figure 11 EPR spectra of TM in the reaction of WLN4. Samples were prepared on 1 mM WLN4 and 4.5 mM TM in the presence of 4.5 mM reducing agent. Spectra were recorded at 140 K with the parameters: microwave power 1 mW; microwave frequency 9089 MHz; sweep time 60 s; center field 324.3 mT; modulation amplitude 0.35 mT. The peak at g = 1.976 shows the feature signal of Mo(V).

Supplementary Tables

Supplementary Table 1 Data Collection and Refinement Statistics

	TM-WLN4[a]	TM-WLN4[b]
**Data collection**		
Space group	$C222_1$	$C222_1$
Unitcell parameters		
a, b, c (Å)	46.27, 87.74, 78.44	46.11,87.58 ,77.30
α, β, γ (°)	90.00, 90.00, 90.00	90.00, 90.00, 90.00
Resolution range (Å)	50.00–1.60 (1.66–1.60)[c]	50.00–2.10 (2.18–2.10)
Wavelength (Å)	1.37906	0.97776
Unique reflections	20975	9508
Completeness (%)	97.8 (83.3)	95.9(97.4)
Overall I/σ(I)	24.4 (4.0)	10.6 (2.1)
Multiplicity	8.0 (4.8)	3.4 (2.7)
Rsym (%)[d]	6.4 (29.2)	8.3 (37.8)
**Refinement**		
Resolution range (Å)	43.87-1.6	43.791-2.1
$R_{work(\%)}$[e]	16.52	17.44
$R_{free\ (\%)}$[f]	19.54	21.14
RMSD[g] bond lengths (Å)	0.016	0.010
RMSD bond angles	1.622	1.404
**Average B-factors (Å^2)**		
Protein	22.21	37.70
Mo	19.27	31.72
S	49.24	29.58
O	16.86	20.78
Water	36.78	44.23
**Ramachandran plot**		
Most favored regions (%)	100	99.25
Allowed regions (%)	0	0.75
Outliers regions (%)	0	0
**PDB code**	6A71	6A72

[a] The crystal prepared from Cu-WLN4

[b] The crystal prepared from apo-WLN4

[c] The values in parentheses refer to statistics in the highest shell.

[d] $R_{sym} = |I_i - \langle I \rangle| / |I_i|$ where I_i is the intensity of the ith measurement, and <I>is the mean intensity for that reflection.

[e] $R_{work} = |F_P - F_{P(calc)}| / F_P$.

[f] R_{free} was calculated with 5.1% of the reflections in the test set.

[g] Statistics for the Ramachandran plot from an analysis using MolProbity.

Supplementary Table 2 MALDI-TOF signals observed in the dimerization of MBDs*

	Dimerization	**Observed *m/z***	**Calculated *m/z***
**a**	WLN4-TM-WLN4	15588	15607
**b**	WLN6-TM-WLN6	16134	16178
**c**	WLN4-TM-WLN4	15600	15607
	WLN4-TM-WLN6	15892	15893
**d**	WLN4-TM-WLN4	15531	15607
	WLN4-TM-WLN5	15986	16045

* Data are from Supplementary Figure 4

Supplementary Table 3 ESI-MS signals observed in the reaction of WLN4 with cisplatin.

	**Composition***	**Observed *m/z***	**Calculated *m/z***
1	WLN4	1535.60	1536.02
2	WLN4+NH_4	1539.16	1539.34
3	WLN4+2NH_4	1542.56	1542.54
4	WLN4+2Na	1544.95	1544.94
5	WLN4+Cu	1548.54	1548.65
6	WLN4+Cu+NH_4	1551.94	1552.05
7	WLN4+Cu+2NH_4	1555.35	1555.45
8	WLN4+Cu+2NH_4+Na	1560.14	1559.65
9	WLN4+[$Pt(NH_3)_2$]	1581.56	1581.56
10	WLN4+[$Pt(NH_3)_2$] +NH_4	1584.76	1584.96
11	WLN4+[$Pt(NH_3)_2$] +2NH_4	1588.36	1588.36
12	WLN4+[$Pt(NH_3)_2Cl$] +Cu+2H_2O	1608.97	1608.64

* +5 charged signals were selected; charges and protons were omitted for clarity.

Supplementary Methods

Protein purification

Cells were harvested by centrifugation at 3005 g for 20 min at 4°C and resuspended in lysis buffer (50 mM Tris, 200 mM NaCl, pH 8.0). The cells were then homogenized by sonication and the lysate was centrifuged at 22324 g for 30 min at 4°C. The resulting supernatants containing target protein fused with N-terminal hexahistidine-tag was subjected to a nickel-nitriloacetic acid (Ni-NTA, QIAGEN) affinity chromatography column pre-equilibrated with lysis buffer. The column was washed with 10 column volumes of washing buffer (50 mM Tris, 200 mM NaCl, 20 mM imidazole, pH 8.0) to remove contaminants and the bound protein was harvested with 10 column volumes of elution buffer (50 mM Tris, 200 mM NaCl, 200 mM imidazole, pH 8.0). The tag of eluted proteins was cleaved by enzymes (TEV for WLN4, MNK3 and the mutants of WLN1234, and SUMO for WLN1234 and mutants of WLN456) at 16°C. Some additional residues are remaining on the N-termini after enzyme cleavages; a SNA sequence was left on WLN4 and MNK3 after TEV cleavage, and a GHMASMTGGQQMGRGS sequence was left on the mutants of WLN1234. Then the targeting protein was purified by AKTA purifier equipped with Superdex 75 gel filtration column pre-equilibrated with running buffer (50 mM Tris, 200 mM NaCl, pH 8.0). The targeting protein was concentrated to 5 mL and was injected into the gel filtration column, and the fraction corresponding to the target proteins were collected for further use.

Preparation of TM bound protein

The Cu-WLN4 was prepared by the incubation of the purified WLN4 protein with 1.5-molar ratio of $[Cu(CH_3CN)_4]ClO_4$ for 15 min at room temperature. The TM-bound proteins were prepared by the reaction of Cu-WLN4 or apo-WLN4 with 3-fold molar ratio of TM for 3 h at 37°C. The TM-WLN4 samples were further purified by AKTA purifier using a HiLoad 10/300 Superdex 75 pg size exclusion column (GE healthcare) equilibrated with SEC buffer (50 mM MES, 150 mM NaCl, pH 6.0). Protein peak was collected and concentrated for further use (Supplementary Figure 3a).

Structure determination and refinement

The structures of TM-WLN4 complex were determined by molecular replacement using Phaser [1] implemented in the CCP4i [2] program package. A Crystal structure of the adduct HAH1-Cd(II)-MNK1 (PDB Code:3CJK) was used as the search model. After several rounds of refinement using REFMAC5 [3] and COOT [4], the structure of TM-WLN4 from Cu-WLN4 was refined to 1.6 Å with a final R_{work} of 16.5% (R_{free}=19.5%). The structure of TM-WLN4 from apo-WLN4 was refined to 2.1 Å with a final R_{work} of 17.4% (R_{free}=21.1%). The final model quality was analyzed by PROCHECK[5]. The data collection and structure determination statistics are listed in Supplementary Table 1. All structure figures were prepared using PyMOL [6].

ICP-MS

After the reaction of Cu-WLN4 with different ratio of TM, copper was separated with ultrafilteration (cutoff 3 kDa). The flow through was collected and digested with aqua regia (3:1 HCl: HNO_3). The mixture was heated on the hot plate until all aqua regia dried out

thoroughly; then 4 mL Milli-Q water was added to dissolve metal ions. The flow through of Cu-WLN4 in the absence of TM was used as a control. The metal content was determined using Plasma Quad 3 inductively coupled plasma mass spectrometer (Thermo VG Elemental, UK, USA).

Gel electrophoresis

Gel electrophoresis was analyzed by Tricine-SDS PAGE with 15% gel. The voltage was set at 100 V in the first 30 min, and then increased to 150 V. The gel was stained with Coomassie Brilliant Blue R-250 and destained using ethanol and acetic acid (v/v 9:2). Images were recorded on a Tanon-1600 digital gel image analysis system.

Circular dichroism

50 μM protein samples were prepared in 2 mM Hepes, pH 6.0. The data was recorded on a Jasco J-720 CD chiroptical spectrometer at 25°C. Far-UV CD spectra (200-260 nm) were collected with a scan speed of 100 nm/ min under nitrogen atmosphere.

EPR spectroscopy

1 mM WLN4 was incubated with 4.5 mM reductant for 10 min, then mixed with 4.5 mM TM. The resulting mixture was transferred into EPR tube and frozen in liquid N_2. X-band spectra were recorded on a JES-FA200 spectrometer at 140K.

ESI-MS

ESI-MS spectroscopy was applied for analysis of the reaction of WLN1234 with TM. The measurements were performed on Waters Xevo G2-Xs QTof mass spectrometer. Data were recorded in an m/z range of 100 to 4000 in positive mode. Spectra were analyzed using Masslynx V4.1.

Supplementary References

1 McCoy, A. J. *et al.* Phaser crystallographic software. *Journal of Applied Crystallography* **40**, 658-674, doi:10.1107/S0021889807021206 (2007).

2 Collaborative Computational Project, N. The CCP4 suite: programs for protein crystallography. *Acta Crystallographica. Section D, Biological Crystallography* **50**, 760-763, doi:10.1107/S0907444994003112 (1994).

3 Murshudov, G. N., Vagin, A. A. & Dodson, E. J. Refinement of macromolecular structures by the maximum-likelihood method. *Acta Crystallogr D* **53**, 240-255, doi:Doi 10.1107/S0907444996012255 (1997).

4 Emsley, P. & Cowtan, K. Coot: model-building tools for molecular graphics. *Acta Crystallogr D* **60**, 2126-2132, doi:Doi 10.1107/S0907444904019158 (2004).

5 Laskowski, R. A., Macarthur, M. W., Moss, D. S. & Thornton, J. M. Procheck - a Program to Check the Stereochemical Quality of Protein Structures. *Journal of Applied Crystallography* **26**, 283-291, doi:Doi 10.1107/S0021889892009944 (1993).

6 DeLano, W. L. The PyMOL Molecular Graphics System. *DeLano Scientific, San Carlos, CA, USA.* (2002).
